# Acute Cyanide Toxicity and Emergency Management in a Goldsmith: A Case Report

**DOI:** 10.7759/cureus.68254

**Published:** 2024-08-30

**Authors:** Mythily Rao Ponaganti, Pavan Tudi

**Affiliations:** 1 Internal Medicine, KIMS (Krishna Institute of Medical Sciences) Sunshine Superspeciality Hospital, Hyderabad, IND

**Keywords:** mitochondrial enzymes, cyanide toxicity, high anion gap metabolic acidosis, hyperlactatemia, antidote

## Abstract

Acute cyanide toxicity is very rare but it is almost always associated with fatal outcomes. Here we describe the case of a 43-year-old healthy male who worked in a jewelry factory and presented with acute cyanide toxicity. He was successfully managed with all the supportive measures and an appropriate antidote kit containing amyl nitrite, sodium nitrite, and sodium thiosulfate. We also describe the relevant importance of knowing the history of easy access to cyanide as a part of the patient's profession, the critical nature of the patient at presentation, as well as the efforts needed to procure the antidote.

## Introduction

As mentioned by Graham and Traylor, cyanide is a rapidly acting cytotoxic substance that is traditionally known as a poison [1]. Cyanide toxicity can be acute or chronic. Various routes of exposure include inhalation, ingestion, and, less commonly, dermal absorption. Oral ingestion of cyanide salts is not so common and it could result from accidental, homicidal, or suicidal intentions.

Cyanide salts are widely used in the jewelry industry but are not commonly available by the general population in retail markets. According to Naik's study, there have been several reports of fatal and near-fatal cases of cyanide poisoning among jewelry industry workers from India and other countries [2]. As mentioned in a study from northern India, the population at risk includes people working in manufacturing companies related to photography, electroplating, chemicals, and plastics and those involved in the polishing of gold, silver, and diamond jewelry [3].

Cyanide is rapidly absorbed and symptoms begin within a few seconds of exposure. The rapidity with which symptoms start depends on the route and amount of exposure and death can occur in less than 30 minutes. The average lethal dose for potassium cyanide is about 250 mg which is most commonly used for gold stripping.

Here, we report a case of acute cyanide intoxication due to suicidal ingestion of potassium cyanide of unknown quantity. The patient was managed with timely supportive measures like oxygen supplementation, volume resuscitation, inotropic support, and electrolytes monitoring and early procurement and administration of an antidote kit, which led to a successful outcome without any long-term complications.

## Case presentation

A 43-year-old healthy male, who is a goldsmith by occupation and works in a jewelry factory was brought to the emergency department (ED) by his wife with an alleged history of ingestion of white crystals (unknown compound) of an unknown quantity. His wife noticed two episodes of vomiting immediately following ingestion. He was brought to the ED approximately 15 minutes after the ingestion of the compound. At the presentation, the patient was frothing from the corners of the mouth and was gasping with tachycardia. He was immediately intubated and mechanically ventilated. On ventilator support, he initially required a 40% fraction of inspired oxygen (FiO2) to maintain adequate saturation. 

His blood pressure was 100/80 mmHg in the right upper arm, pulse rate was 146 beats per minute with regular rhythm and normal volume, oxygen saturation was 100% at 40% FiO2, body temperature was 98.7F, and pupils were 2 mm, reacting to light. Arterial blood gas (ABG) analysis revealed high anion gap metabolic acidosis with elevated lactate levels, as shown in Table 1. ECG showed sinus tachycardia. Co-oximetry was done which showed normal levels of carboxyhemoglobin (1.2%) and methemoglobin(3.9%). 

**Table 1:** ABG at presentation showing high anion gap metabolic acidosis with hyperlactatemia ABG: arterial blood gas; pO2: partial pressure of oxygen; pCO2: partial pressure of carbon dioxide; HCO3-: bicarbonate

ABG Components	Calculated Value	Reference Value
pH	7.13	7.35-7.45
pO2	79 mmHg	83-108 mmHg
pCO2	14 mmHg	32-48 mmHg
HCO3-	7 mEq/L	22-26 mEq/L
Lactate	15 mmol/L	0.5-1 mmol/L
Sodium	142 mmol/L	135-145 mmol/L
Potassium	3.75 mmol/L	3.5-5.5 mmol/L
Chloride	102 mmol/L	96-106 mmol/L
Anion gap	33 mmol/L	10-12 mmol/L

Meanwhile, a coworker of the patient gave a history of using potassium cyanide for diamond polishing at his workplace and confirmed it to be a cyanide compound. Ryle's tube was inserted immediately and gastric lavage was done with normal saline and 50 grams of activated charcoal and he was shifted to ICU for further management. The patient was given all the appropriate supportive measures and was monitored in the ICU. But in an hour his condition gradually deteriorated leading to hypotension and he was started on inotropic support with noradrenaline and required a gradual increase in FiO2 levels. 

As specific treatment with cyanide antidotes is very crucial for the survival of the patient, which was not readily available at our pharmacy, we reached out to other hospital pharmacies. However, it was not available at many other hospital pharmacies and even at his workplace, the jewelry factory; hence, it was procured directly from a pharmaceutical company within three hours. By then the patient had started desaturating with oxygen saturations of 80% even at 100% FiO2. The antidote kit containing three compounds was administered as per the instructions available in the kit. The compounds included amyl nitrite inhalant durules which were given via endotracheal tube for 20 seconds with intermittent intervals of 15 seconds of 100% FiO2. A total of 10 rules of amyl nitrite were administered, which was followed by intravenous sodium nitrite 300 mg for two minutes at 5 ml/minute (10 ml), which was then followed by a slow intravenous infusion of sodium thiosulfate 25 mg (50 ml).

The patient's general condition started improving within 30 minutes of antidote administration. Repeat ABG analysis after an hour showed drastic improvement in acidosis and a decrease in lactate levels. The patient regained consciousness but was drowsy initially. As saturation levels improved, FiO2 was tapered very slowly over the next day with continuous monitoring of vital parameters and ABGs. Routine serum chemistry revealed normal values without any organ dysfunction. The patient was further stabilized with decreasing levels of inotropic support and oxygen support and he was able to maintain adequate oxygen saturation with minimal FiO2 of 30%.

After almost 48 hours of admission, the patient was off the inotropic support and was able to meet the extubation criteria. ABG analysis showed normal pH levels and lactate levels. Repeat ECG was normal. After a successful spontaneous breathing trial, he was extubated on day three of admission, following which he required minimal oxygen support via a face mask.

The patient's sensorium showed marked improvement and he was fully oriented by the fourth day and oxygen support was stopped. He started on oral clear liquids which he tolerated well, then followed by normal dietary intake. He was counseled along with family members by a psychiatrist and later discharged home in a stabilized condition. The patient was followed up after a week with a complete blood count and comprehensive metabolic panel, both of which were normal. His vital signs were stable, and he was able to resume his daily routines.

## Discussion

Cyanide is a rapidly acting cytotoxic substance that is traditionally known as a poison with a very low lethal dose. As discussed by Jones, hydrogen cyanide was first isolated from Prussian blue dye in 1786 and cyanide was first extracted from almonds around 1800 [4]. Cyanide exists in gaseous, liquid, and solid forms. Hydrogen cyanide (HCN), also known as prussic acid, is a volatile liquid that boils at 25.6° C (78.1° F). Potassium and sodium cyanide salts are water-soluble, whereas mercury, copper, gold, and silver cyanide salts are poorly water-soluble. 

Most of the cyanide poisonings occur from accidental exposure or intentional ingestion of cyanide-containing salts. Modes of exposure are usually inhalation of smoke, containing hydrogen cyanide gas, absorption from contaminated skin and eyes, oral ingestion of cyanide-containing salts, apple seeds, or cassava beans, etc., and less commonly from intravenous administration of sodium nitroprusside in ICU patients. As reported in Graham and Taylor's study, fire is the most common source of cyanide exposure in industrialized countries such as the United States [1]. Suicidal exposures are rarely reported and account for only 11 out of 200 cyanide poisoning cases. According to the American Association of Poison Control Center's Toxic Exposure Surveillance System, four of the 163 cyanide exposure cases in 2021 were fatal.

Susceptible populations include those working in industries related to photography, electroplating, chemicals, and plastics and those involved in the polishing of gold, silver, and diamond jewelry. The relatively easy access to the cyanide-containing salts and their direct handling as a part of the occupation can lead to accidental exposure or suicidal deaths. As discussed in our case, the patient worked in a jewelry factory where he had easy access to potassium cyanide which is used for gold and diamond polishing. Another case reported described accidental exposure while cleaning an electroplating chamber in a handicraft industry, which led to the death of three men before reaching the ED with the autopsy revealing it to be because of the complications associated with cyanide poisoning [5]. Fatality and outcome depend majorly on the type and amount of exposure, and to a minor extent on liver function, preexisting cyanide levels, and the presence of B12 levels as mentioned by Baud [6]. Death can occur very quickly due to inhalation of cyanide compared to other modes of exposure. In our case, the patient has intentionally ingested potassium cyanide of an unknown quantity and the lethal dose is 250 mg

As discussed by Way, cyanide is a rapidly acting deadly poison and attaches itself to cytochrome oxidase, which is a ubiquitous metalloenzyme [7]. The cyanide makes the enzyme inactive by reacting with ferric iron, uncoupling oxidative phosphorylation, and inhibiting cellular respiration even in the presence of adequate oxygen levels. As a result, cellular metabolism shifts from aerobic to anaerobic with the production of lactic acid. As in our case, the ingested potassium cyanide reacts with acidic contents of the stomach and gets converted to HCN, which readily enters cells and inhibits the mitochondrial electron transport chain.

The onset of symptoms and the severity depends on the mode of toxicity and amount of exposure. As in the study by Hendry-Hofer et al., exposure to cyanide via the inhalation route results in symptoms within seconds of exposure, whereas symptoms following ingestion occur in minutes to hours [8]. The symptoms associated with low-dose exposures are mild and not life-threatening and they include headache, dizziness, mild confusion, nausea, vomiting, and abdominal cramping. Large-dose exposures are life-threatening and are associated with worse outcomes and faster deterioration leading to dyspnea, respiratory depression, apnea, hypotension, arrhythmias, coma, seizure, and death. Our patient initially had vomiting later followed by respiratory symptoms and hypotension requiring ventilation and vasopressor support.

Diagnosing acute cyanide intoxication is very difficult. It is almost always based on circumstantial evidence and presenting signs and symptoms. As Parker-Cote et al.'s study mentioned, the presence of high anion gap metabolic acidosis with hyperlactatemia and increased oxygen levels in venous blood points towards cyanide poisoning [9]. Although serum cyanide levels can be done to confirm the toxicity, it is not so useful for the initial diagnosis and management of the patient. In the case of inhalation injury cyanide poisoning should be differentiated from other poisonings like carbon monoxide poisoning and also methemoglobinemia, which is characterized by the presence of a saturation gap. In our case, the diagnosis was initially suspected by his clinical presentation and his profession with a history of ingestion of white crystals, later confirmed by a coworker.

As acute cyanide toxicity is very dangerous and life-threatening, the most crucial initial management is to stabilize patients' airways, breathing, and circulation. Decontamination also plays a vital role in the prevention of early deterioration and poor outcomes. Patients must be removed from the source of exposure, their clothing removed and discarded appropriately, and gastrointestinal decontamination must be administered as early as possible. Although lab studies have demonstrated poor binding of activated charcoal to cyanide, in animal studies, mortality decreased when subjects were given activated charcoal [1]. Therefore aggressive treatment is suggested with a single dose of activated charcoal of 50 g in adults and 1 g/kg, up to a maximum of 50 g in children, to be given. In our case, the patient was given activated charcoal, and gastric lavage was done quickly in ED, which might have contributed to the delay in desaturation and sufficient time to procure the antidotal kit.

Definitive management of cyanide toxicity is to administer an antidote kit, which usually contains amyl nitrite inhalants, sodium nitrite, and sodium thiosulfate injections. As Mannaioni et al.'s study suggests, timely and appropriate use of antidotes for cyanide intoxication prevents death even in older and diabetic patients [10]. The most widely used antidotes worldwide are nitrites with thiosulfates. Nitrite compounds in the antidote kit convert Hb(Fe2+) to methemoglobin (Fe3+), which then scavenges cyanide. On the other hand, sodium thiosulfate serves as a sulfur donor thereby increasing the rate of rhodanese-catalyzed transformation of cyanide to thiocyanate, which is excreted by kidneys. Apart from conventional antidote kits, hydroxocobalamin is also widely used in cyanide poisoning which when combined with cyanide forms cyanocobalamin which is a stable compound excreted by kidneys. Many studies consider treatment with sodium thiosulfate and hydroxocobalamin efficacious with little inherent toxicity, unlike nitrite compounds [11-13]. In our case, we administered an antidote kit containing nitrites and sodium thiosulfate after procuring it from a pharmaceutical company, which resulted in drastic improvement in the patient's vital parameters and clinical condition, finally leading to a positive outcome.

## Conclusions

Patients arriving at the ED with accidental or deliberate ingestion of an unknown compound in unstable conditions with high anion gap metabolic acidosis and hyperlactatemia should always have cyanide toxicity included as an important differential diagnosis. The diagnosis is almost always based on circumstantial evidence and presenting features. The most important clue to the diagnosis is severe metabolic acidosis with elevated lactates and arterialization of venous blood gases. Diagnostic studies are time-consuming and death can occur in less than 30 minutes based on route and amount of exposure. As it is associated with a grave prognosis and high fatality, it is always better to initiate the management like the removal of clothes and gastric lavage as early as possible. It would be prudent for the ED of each hospital to be well equipped with a life-saving antidote kit and also for timely management of acute cyanide toxicity cases for better outcomes.
